# Gaps in the evidence underpinning high-risk medical devices in Europe at market entry, and potential solutions

**DOI:** 10.1186/s13023-023-02801-7

**Published:** 2023-07-25

**Authors:** Frank Hulstaert, Céline Pouppez, Célia Primus-de Jong, Kathleen Harkin, Mattias Neyt

**Affiliations:** 1 Administrative Centre Botanique, Belgian Health Care Knowledge Centre (KCE), Doorbuilding (10th floor), Boulevard du Jardin Botanique 55, Brussels, B-1000 Belgium; 2 Centre for Health Policy and Management, Trinity College Dublin, 3-4 Foster Place, Room 0.18, Dublin, Ireland

**Keywords:** Medical devices, Evidence gaps, CE marking, Reimbursement, Declaration of Helsinki, Comparator, Study design, Comparative evidence

## Abstract

**Aim:**

To determine the level of evidence for innovative high-risk medical devices at market entry.

**Methods:**

We reviewed all Belgian healthcare payer (RIZIV-INAMI) assessor reports on novel implants or invasive medical devices (n = 18, Class IIb-III) available between 2018 to mid-2019 on applications submitted for inclusion on their reimbursement list. We also conducted a review of the literature on evidence gaps and an analysis of relevant legal and ethical frameworks within the European context.

**Findings:**

Conformity assessment of medical devices is based on performance, safety, and an acceptable risk-benefit balance. Information submitted for obtaining CE marking is confidential and legally protected, limiting access to clinical evidence. Seven out of the 18 RIZIV-INAMI assessor reports (39%) included a randomized controlled trial (RCT) using the novel device, whilst 2 applications (11%) referred to an RCT that used a different device. The population included was inappropriate or unclear for 3 devices (17%). Only half of the applications presented evidence on quality of life or functioning and 2 (11%) presented overall survival data. Four applications (22%) included no data beyond twelve months. The findings from the literature demonstrated similar problems with the study design and the clinical evidence.

**Discussion and conclusions:**

CE marking does not indicate that a device is effective, only that it complies with the law. The lack of transparency hampers evidence-based decision making. Despite greater emphasis on clinical benefit for the patient, the provisions of the European Medical Device Regulation (MDR) are not yet fully aligned with international ethical standards for clinical research. The MDR fails to address key issues, such as the lack of access to data submitted for CE marking and a failure to require evidence of clinical effectiveness. Indeed, a first report shows no improvement in the clinical evidence for implantable devices generated under the MDR. Thus, patients may continue to be exposed to ineffective or unsafe novel devices. The Health Technology Assessment Regulation plans for Joint Scientific Consultations for specific high-risk devices before companies begin their pivotal clinical investigations. The demanded comparative evidence should facilitate payer decisions. Nevertheless, there is also a need for legislation requiring comparative RCTs assessing patient-relevant outcomes for high-risk devices to ensure implementation, including development and implementation of common specifications for study designs.

## Background

Healthcare has an ethical dimension as it aims to prevent and alleviate human suffering. Healthcare has also developed into an important economic sector, with sales of services and products. Total European Union (EU) expenditure on healthcare (public and private) amounts to around €1.3 trillion annually, including €220 billion for medicinal products and €100 billion for medical devices. Healthcare spending represents about 10% of EU GDP [1].

This paper is based on a report published by the Belgian Healthcare Knowledge Centre (KCE) that investigates whether the clinical trial data for medicinal products and medical devices leading to market entry also meet the needs of health technology assessment (HTA) bodies and healthcare payers for the assessment of added therapeutic benefit [2]. This paper covers the following research questions: 1. What are the European legal requirements and (international) ethical standards for medical devices regarding clinical evidence? 2. What clinical evidence is available to support manufacturers’ claims of added therapeutic value for innovative medical devices upon market entry or when first seeking reimbursement?

## Methods

The research incorporated three different studies: an analysis of the international ethical frameworks and European legislation governing medicines and medical devices; an examination of assessors’ reports on applications submitted to the Belgian public healthcare payer, the National Institute for Health and Disability Insurance (RIZIV-INAMI); and a review of the literature on reviews of groups of medicines or medical devices examining their evidence base. The draft report was shared with forty-one experts who provided their feedback via email and during three expert group meetings (7 from legal, ethics, or regulatory backgrounds; 22 payers/HTA personnel; 12 clinicians and/or researchers from academia, charity, and consumer organisations). This feedback was incorporated into the final draft report and shared with industry representatives requesting their comments, eight of whom joined a stakeholder meeting to provide their feedback prior to publication of the report. The final draft report was shared with three external experts for peer review and comment (validation). The policy recommendations were reviewed and accepted by the Board of the KCE, which is composed of representatives of the Belgian government as well as other healthcare stakeholders.

In this paper, the focus is on innovative high-risk medical devices (Class IIb and III). Compared with pharmaceuticals, some medical devices present additional factors that affect their clinical assessment. For implantable devices, these factors include the surgical procedure itself, the learning curve, the outcome assessment, a possible volume-outcome relationship, and difficulty in determining the optimal time at which to conduct the randomized controlled trial (RCT) - all elements that need to be taken into account during evidence generation [3–5].

Legal and ethical rules were examined since the European legal context is evolving, with the recent medical device regulation (MDR) [6] and a new regulation on health technology assessment (HTAR) [7]. We specifically examined the evidence and transparency requirements, ethically and legally, for medical research and medical devices being placed on the European market.

We reviewed all Belgian healthcare payer (RIZIV-INAMI) assessor reports available between 2018 to mid-2019 for eighteen novel implants or invasive medical devices (all Class IIb-III) submitted for inclusion on the reimbursement list (Royal Decree of 25 June 2014) [8]. Gaps in the evidence that were identified and explicitly reported as an issue in the assessor’s reports were extracted and arranged thematically according to the PICOTS framework (patient/population, intervention, comparator, outcome, timing – duration of treatment and follow-up, setting and study design)[9] – evidence that is crucial for clinical and reimbursement decision-making. The information obtained from the literature review and from the feedback received from HTA experts was also analysed according to each of these themes.

A systematic search strategy was developed using key words and controlled vocabulary terms for the concepts of ‘medical devices’ AND ‘evidence/evidence gaps’ AND ‘reimbursement/funding or market entry’ AND ‘Europe’. The searches were performed in PubMed, EMBASE, and the Cochrane Library in 2019 (17 September-17 December), revised in January 2021, and updated in August 2021. Searches were limited by date (published after June 1st 2011) based on the last search of a previous KCE report on this subject. Titles, abstracts, and selected full texts were screened in phases against the inclusion/exclusion criteria independently by two reviewers (FH and CPdJ). Reports were eligible for inclusion if they reviewed the evidence available for groups of high-risk medical devices at the time of market entry or application for reimbursement in the EU. The final update was screened by just one reviewer (CPdJ). Results were compared and differences were resolved through discussion and consensus. A narrative synthesis of findings regarding the quality of the evidence base and gaps identified is presented in the KCE report, with further details available on p.85–87; 152–208 [2].

This article focusses primarily on the legal/ethical analyses. We also discuss the analysis of the reimbursement applications in the context of the findings of the literature review.

## Findings

### European legislation and international ethical standards for medical devices

#### Evidence requirements

In Europe, the regulatory framework for medical devices, including high-risk medical devices, differs greatly from the regulatory system for medicinal products. It also differs significantly from the Food and Drug Administration’s (FDA’s) approach to regulating medical devices in the United States (US).

It is standard practice to conduct an RCT of a novel medicine during its development to demonstrate its clinical safety and efficacy, with discussions focussing on the appropriate choice of the comparator and study endpoints. However, despite a positive trend towards more RCTs for medical devices [10], this is still not the standard approach to the clinical development of medical devices in Europe.

In the EU, medical devices are subject to Regulation 2017/745 (MDR) [6], which was enacted in May 2017 and, following a year’s postponement as a result of the global COVID-19 pandemic, came into effect in May 2021. It replaces the previous Medical Device Directives (MDDs) governing active implantable devices and general medical devices, but excludes in-vitro diagnostic devices, which are governed by separate legislation. In the meantime, transitional arrangements allow devices certified under the MDDs to remain on the market, if their certificates have not already expired. This transition period has recently been extended, enabling most Class III devices (meeting specified requirements) to remain on the market until May 31, 2027 [11].

Both the MDDs and the MDR require clinical studies (clinical investigations) for implantable medical devices. However, there are exceptions. These include implants that are classified as ‘sutures, staples, dental fillings, dental braces, tooth crowns, screws, wedges, plates, wires, pins, clips and connectors’,(MDR, Article 61.6) and, with certain preconditions, devices that are modifications of devices already legally placed on the market may also be exempted (MDR, Article 61.4-5) [6]. However, even where clinical investigations are required, these need not necessarily be RCTs, nor do they have to assess (comparative) clinical effectiveness. Furthermore, there are no specific requirements imposed regarding the design, scope, or duration of the study, nor is the ethical review standardised as each member state is responsible for organizing their own system for reviewing proposed study designs and their compliance with ethical requirements.

“CE marking” (Conformité Européenne) is a mark that indicates (though it doesn’t guarantee) compliance with the relevant legal requirements. The Commission notes that “a CE marking does not indicate that a product have been approved as safe by the EU or by another authority” [12]. Manufacturers must affix the CE marking to their devices following an assessment of their compliance with the EU legal requirements for medical devices in a process called conformity assessment. This conformity assessment procedure and the CE marking are required in order to gain access to the EU market. For high-risk medical devices compliance is assessed and certified by Notified Bodies, often for-profit organisations, with which the manufacturer has a contract. Notified Bodies are designated to perform conformity assessments by the government of the member state where they are located (through a designating authority) [13].

In order to be deemed compliant, medical devices must “satisfy general requirements” with regard to their safety and performance in normal use, and show that their benefit/risk ratio is acceptable. Note that, in contrast to the US situation, the demonstration of effectiveness is not a requirement in Europe [14]. In the US, the effectiveness of innovative high-risk devices must generally be proven, for example, with an RCT comparing the clinical effects of the medical device with a relevant comparator or with a sham procedure [14].

The CE marking system remains largely based on an unsubstantiated trust that the necessary evidence of safety and effectiveness will be provided after the product is placed on the market [15, 16]. Thus, the clinical data required for approval of the CE marking by Notified Bodies are rather limited, even for high-risk devices. Indeed, both the MDDs and the MDR allow clinical data derived from a literature review on the safety and performance of another device to be used for the approval of a new device if it is considered to be ‘equivalent’ (MDR Article 2.48) [6]. The concept of equivalence was not previously defined under the MDDs [17], and has been open to interpretation, however, the MDR has rectified this shortcoming by providing a legal definition of equivalence that takes technical, biological and clinical characteristics into account (MDR, Article 2, Annex XIV.3) [6]. Nevertheless, the concept of equivalence explains why many medical devices come onto the European market on the basis of a literature review of similar devices rather than being based on direct clinical data (i.e. no clinical studies of their own) [18].

The new MDR has tightened the rules somewhat by requiring that “clinical benefit” be demonstrated in addition to “clinical performance”. “Clinical benefit” is defined as the positive impact of a device on the health of an individual, expressed in terms of a meaningful, measurable, patient-relevant clinical outcome(s), including outcome(s) related to diagnosis, or a positive impact on patient management or public health (MDR, Article 2.48) [6]. In addition, the clinical evaluation must take other available treatment options into account, however, comparative evidence is not legally required (MDR, Article 61.3(c)) [6]. It is important to note that ‘clinical evaluation’ is not a clinical trial as might be supposed, but rather an assessment of all the clinical data from any relevant source, including literature on similar devices (MDR, Article 2.44) [6]. Thus, a clinical evaluation may not include any data from a clinical trial, comparative or otherwise, of the actual device.

The MDR also introduces the possibility for the EU regulatory authorities and Notified Bodies responsible for high-risk devices to have access to independent Expert Panels via a procedure called the *clinical evaluation consultation procedure (CECP)* (MDR, Article 54) [6]. The list of opinions provided under the CECP are made publicly available on the European Commission’s website [19]. In addition, provisions are made for these experts to also be consulted voluntarily by an individual manufacturer, although the introduction of this latter option is still only in the pilot phase (MDR, Article 61.2; Article 106.10(e)) [6]. The HTAR, for specific medical devices, provides the possibility of an early dialogue or Joint Scientific Consultation (JSC) with the European HTA bodies before the start of the pivotal clinical investigations as well as a Joint Clinical Assessment (JCA) that feeds into the HTA at a national or regional level.

#### Role of national competent authorities and ethics committees in shaping the evidence

A clinical investigation under the scope of the MDR can only be conducted if authorised by a national competent authority (NCA) and if no negative opinion has been issued by a research ethics committee (REC) with regard to the clinical investigation (MDR, Article 62.4) [6].

Article 71 of the MDR requires that the NCAs assessing applications for clinical investigations must review, amongst other things, the reliability and robustness of the data generated in the clinical investigation, taking account of statistical approaches, the design of the investigation and methodological aspects, including sample size, comparator and study endpoints. This assessment must respect the strict authorisation process timelines applicable under the MDR for each clinical investigation. Provided the member state national REC has not issued a negative opinion, authorisation must be provided immediately following application validation for Class IIb devices and within 45 days of a validated application for Class III devices (or 65 days if expert panels are consulted), with designated pauses if additional information is requested (MDR, Article 70.7(b) [6].

However, the MDR does not specify how the RECs should conduct reviews of trial applications. Indeed, under the MDR, it is left to the member state where the clinical investigation is to be conducted to decide who is the appropriate authority to assess the clinical investigation application and to define the extent of the ethics review required.

According to the Declaration of Helsinki, a control group receiving no treatment, a sham procedure, or a placebo may be justifiable as a comparator *only if* no proven treatment or standard of care is available or where it is required for compelling and scientifically sound methodological reasons as the only means to determine the efficacy or safety of an intervention [20]. If this is the case, patients who receive no intervention/treatment, a placebo/sham procedure, or any intervention less effective than the best proven one must not be subjected to additional risks of serious or irreversible harm as a result of not receiving the best proven treatment. This standard should guarantee that clinical studies do not expose participants to unjustified risks and burdens no matter what other positive features they might appear to have.

#### Transparency

In contrast to medicinal products in Europe and medical devices entering the US market, clinical data on medical devices previously did not have to be made publicly available upon entry to the EU market. Whilst the MDR still maintains confidentiality of the data submitted for CE marking, nevertheless, its introduction is expected to improve transparency to a certain extent. It requires manufacturers of the highest-risk devices to provide a summary of the most important safety and performance aspects, as well as the results of the clinical evaluation of their devices, in a publicly accessible document, the Summary of Safety and Clinical Performance (SSCP) (MDR, Article 32.1) [6]. In particular, this document must contain a description of the device’s place in therapy relative to the existing alternatives (MDR, Article 32.2(d)) [6]. Furthermore, providing misleading information can be cause for legal action [21]. The SSCP is to be made available via the European medical device database, EUDAMED, part of which will be accessible to the general public [22]. When fully implemented, EUDAMED will consist of six modules or registers covering actor registration, unique device identification (UDI) and device registration, notified bodies and certificates, clinical investigations and performance studies, vigilance and market surveillance [22]. Therefore, EUDAMED also promises to increase the transparency of clinical studies on medical devices. Unfortunately, the implementation of this part of EUDAMED is facing years of delay, so it remains unclear how much data and what level of detail will be reported in this database.

#### European evaluation of health technology

The EU HTAR, which has only recently been enacted, is gradually being implemented [7]. The JCA is a centralised HTA assessment procedure to be conducted at the EU level for certain medicinal products and medical devices. It should ensure that the methodologies and procedures applied in an HTA become more predictable throughout the EU. The centralised HTA and the conformity assessment for CE marking will remain two separate frameworks due to their different purposes, but synergies will be created, with mutual exchange of information and better coordination of the timing of the procedures. These methods and processes are being refined under a European service contract with EUnetHTA 21 [23]. The aim is to be operational in 2030 for the JCAs of specific medical devices. In addition, the EU HTAR aims to introduce a procedure enabling an early dialogue between HTA bodies and medical device companies on their clinical trial plans.

**Fig. 1:**
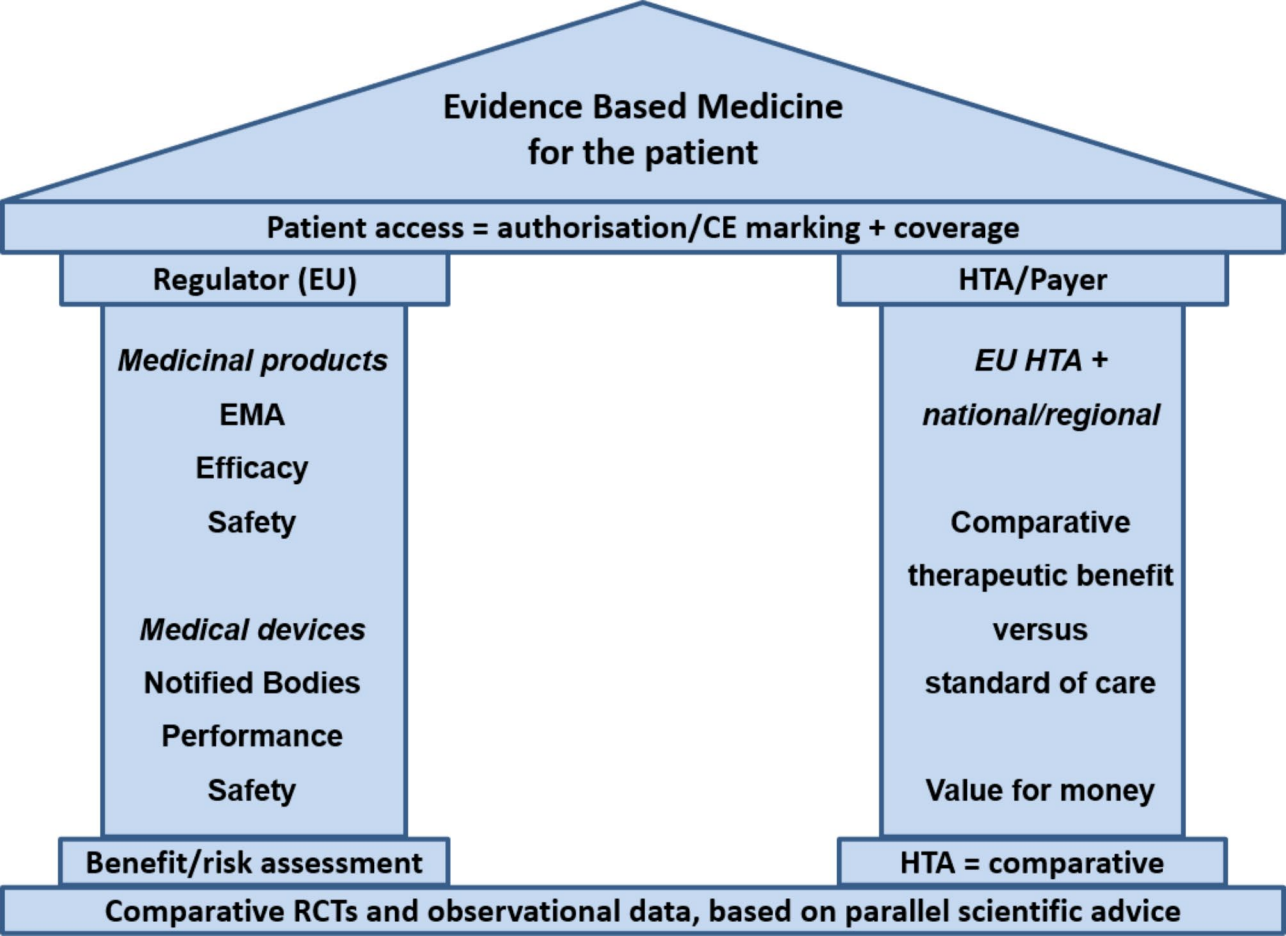
Evidence requirements necessary for patient access to novel health technologies

As depicted in Fig. 1, healthcare payers, and HTA bodies advising them, view the clinical evidence for an innovation from a different perspective to that of the regulators. Health technology assessment is always comparative in nature (comparing the innovation with current standard care). Healthcare payers, and also clinicians, want to see a randomised direct comparison of the new intervention in a representative patient population using patient-relevant outcomes (e.g., overall survival, quality of life, or functional outcomes) versus standard care. This is essential to reliably assess if the new device provides any added therapeutic benefit. In contrast, the focus of the regulators is mainly on the benefit-risk evaluation of a specific intervention, not requiring a direct comparison versus standard care. The regulators’ remit is to *not* prevent products from entering the market so long as they perform as intended and have an acceptable level of risk, whereas, the remit for HTA is to synthesise the necessary evidence to ensure safe, effective, and cost-effective healthcare is provided.

### Evidence gaps in applications for reimbursement of innovative high-risk devices in Belgium

The analysis is based on RIZIV-INAMI assessors’ reports on company applications for medical devices to be included on the reimbursement list of novel implants or invasive medical devices (Royal Decree of 25 June 2014) [8]. Of the 20 consecutive reimbursement applications submitted to RIZIV-INAMI [2018-mid-2019] and provided to KCE for this study 2 were incomplete, leaving 18 for assessment. All 18 devices were invasive and/or implantable, 7 (39%) were intended for the cardiovascular system, whilst the remainder were intended for various other body systems (2 neurological, 2 sensory organs, 2 orthopaedic, 2 surgical instruments).

According to the submission procedure at RIZIV-INAMI companies can indicate a level of added clinical benefit for their device. A claim was made for the highest level of added clinical benefit (Class 1) in 12 applications (67%), whilst for three the claimed level of added clinical benefit was not clearly reported in the application. The device’s intended use suggested a direct impact on quality of life for 11 devices. Six devices could potentially impact overall survival (OS) based on the intended use.

We were given access to the assessor’s reports, which contained little detail, limiting their synthesis solely to aspects reported on. When assessors observed a major weakness in an application they might have based their conclusion on this weakness without going into further detail on all other possible weaknesses. The absence of a comment regarding a specific topic, therefore, does not mean the absence of an issue or problem. The findings from the assessors’ reports are summarized thematically using PICOTS.

#### Patients and setting

Application assessors reported as an issue that the target population for which the reimbursement request was submitted did not match the trial’s patient population for 2 out of 18 devices, and in another application this was unclear. For some target groups no evidence was shown of any additional benefit, and for one device the claimed benefit could not be demonstrated in the RCT’s planned analyses, but in a post-hoc sub-group analysis.

Assessors noted in a few cases that it was unclear for what clinical indication and patient population the device was intended, making it difficult for them to clearly describe the target population.

#### Intervention

In two applications (11%), assessors explicitly expressed concern regarding the fact that the trial data presented in the dossier was on a completely different device to the one for which reimbursement was being sought.

#### Study design, comparator, outcomes and follow-up

Seven applications (39%) included an RCT investigating the innovative medical device and for another two dossiers the RCT tested a device other than the device for which reimbursement was being sought. The lack of RCT data was explicitly mentioned as a problem in one of the assessor reports.

Among the 9 applications containing RCT data, one of these demonstrated inferior efficacy compared with medical treatment; and 4 used surrogate outcomes, one of which showed no difference. Quality of life (QoL) was reported on for 4 (49%) of these devices. Regarding OS, this was either not mentioned as an outcome (n = 4), data were not presented (n = 4) or no effect was shown (n = 1).

In half (9 out of 18) of the device reimbursement dossiers, applicants were seeking reimbursement on the basis of evidence from cohort studies, which suggests that these devices received their CE marking without undergoing an RCT. Eight had at least one prospective cohort, but one reported only on a retrospective cohort comparison. For seven devices (78%) only surrogate outcomes were reported. Only for 2 devices (22%) was QoL reported. Six devices (67%) had no data on OS. One reported on OS, which appeared to be favourable, but there were no comparators.

Finally, for one device a comparison in outcomes between 2 populations was reported rather than between treatments in the population for which reimbursement was being sought.

Overall, more than half (10/18) of the applications contained no data on quality of life or other patient-relevant outcomes, despite all devices being invasive and likely to affect QoL or functioning, either directly or indirectly. In one case this was reported as a major shortcoming by the assessor, and in another application the patient relevant outcome was evaluated in a post-hoc sub-group analysis while the RCT investigated surrogate outcomes.

Overall, QoL outcomes were reported for five out of 18 devices, and some QoL improvement was found in four of these, including for two devices, where pain was assessed and showed improvement. Additionally, three applications contained outcome data on function (1 improved, 1 had small benefit, 1 no effect).

For 5 applications (28%), there was limited (one with 10 patients at 5 years) or no data (n = 4) on outcomes after one year, despite being implantable devices. In two applications, the lack of sufficiently long-term results was explicitly considered a problem by the assessor.

Overall survival was not mentioned in 5 applications (28%), whilst there was explicitly no data available for over half (n = 11). Two reports included data on OS, for one of these it showed no difference, and for the other it appeared to be beneficial, but there was no comparative data.

In conclusion, over 60% (11/18) of the medical device applications submitted to RIZIV-INAMI for reimbursement did not include an RCT on the actual medical device to be reimbursed. Other weaknesses included the reporting of surrogate outcomes only and the short duration of follow-up of patients who had received an implant.

### Evidence gaps in the literature

#### Challenges in identifying the relevant literature

Compared with medicinal products, relatively few studies have reported on the level of evidence and the methodological shortcomings of pre-market clinical evaluations of medical devices in Europe. Medicinal products may have the necessary evidence for a reimbursement decision readily available at the time of regulatory approval, since the clinical development and results of the pivotal trials for each new drug are summarized in a document called a European public assessment report (EPAR). These are available on the European Medicines Agency (EMA) website and are often used as a starting point for studies reviewing the pre-market evidence base underpinning specific groups or categories of medicinal products. In contrast to medicines, there is no such public document yet available summarizing the clinical studies on a given medical device when it enters the European market. Therefore, researchers have had to find other ways to collect and evaluate the evidence base for groups of medical devices. Such reviews are further hampered by strong confidentiality requirements and the absence of a legal obligation under the MDDs to register device trials (assuming they exist). Furthermore, device companies possibly may not make medical device trial results publicly available. Consequently, relatively few studies have quantified the level of evidence and the methodological shortcomings of clinical evaluations of medical devices in Europe.

#### Findings from the literature review

We identified sixteen studies published since June 1st 2011 that reviewed the evidence for groups of high-risk therapeutic medical devices. They each used different methods, and presented their findings in different ways. The majority (11 out of 16) used levels of evidence to grade the quality of the evidence base included in their reviews, applying various different grading systems. Three others described individual elements of study quality, whilst the final one only included RCTs, evaluating their quality using the Cochrane Risk of Bias tool.

Many of the review authors indicated a lack of RCTs for more than half of the devices they reviewed [10, 24, 25, 17, 26, 27, 18, 28–32]. Other issues identified included a poorly described clinical indication or target population [32]; the use of predicate devices [24, 26]; small sample sizes [30, 31]; no patient-relevant study outcomes [10, 17, 28]; or the sole use of surrogate outcomes [28]; and limited long-term data [26]. No evidence was found for some devices [33, 34, 29], whilst some authors found that the evidence for some devices showed them to be no better or less safe/effective than other treatments [27].

## Discussion

### Clinical evidence

Authors noted that manufacturers can use clinical data from other similar medical devices to support applications for CE marking. Indeed, it is only the first generation of an innovative product that has to provide direct clinical evidence on its safety and technical performance, whilst follow-up products can be CE marked “on the basis of their technical equivalence to the first product” [18]. This means that a CE marking is often approved after a simple demonstration of equivalence with another device subject to clinical evaluation. Boudard et al. (2013) highlighted this issue and referenced a French Senate report on the safety of medical devices, which stated that 90% of the medical devices on the French market obtained their CE marking following a demonstration of equivalence [17]. This may have serious consequences because the safety and efficacy of medical devices without clinical trial data remains uncertain and, therefore, these devices could be harmful for patients. In fact, Wild et al. (2014) showed that 24% of cardiovascular devices (7 out of 29) had clear issues after being CE marked, especially devices that had been CE marked via the “substantial equivalence” process [27]. These findings are in agreement with the 2012 FDA report entitled “Unsafe and Ineffective Devices Approved in the EU that were Not Approved in the US” [35].

The main reason for a paucity of pre-market device RCTs seen in the past in Europe is most probably the fact that such RCTs were not needed to obtain a CE marking [36]. Indeed, Heneghan et al. (2017) have shown that it is entirely possible, even in the US, for multiple generations of devices (e.g. transvaginal mesh devices for pelvic organ prolapse) to be approved on the basis of ‘generations’ of equivalence claims [37], and that this is possible even when some of the claimed equivalent devices, the predicates, have been removed from the market due to adverse patient outcomes, device failure, or more stringent evidence requirements. Furthermore, the evidence for this failure is growing [38, 39].

Due to the differences in the European and US regulatory approaches, innovative medical devices are often available more quickly on the European market, but this is on the basis of minimal clinical data, even for implantable devices. For example, this lower level of evidence was seen in the 2008 applications submitted for reimbursement in France [24], and was confirmed in a later report from Austria [27]. In cases where the evidence provided is considered to be insufficient to make a recommendation, HTA bodies in Europe may decide to await the results of an RCT requested by the FDA to demonstrate effectiveness. The issues associated with HTA of high-risk medical devices have been the subject of two KCE reports and related papers [14, 40]. They particularly addressed the lack of sufficient evidence for HTA purposes when a device is CE marked as well as the lack of evidence transparency. Specific issues related to 3D-printed medical devices are covered in a separate KCE report [41].

Where RCTs of medical devices do exist, they are generally poor in quality and multiple shortcomings have been reported, such as the lack of a clearly defined primary endpoint or the absence of a description on the handling of missing data, amongst others [17]. For implants, the study duration is often short compared with their long duration of use, which, as previously mentioned, is an issue for determining whether to provide reimbursement and/or at what price [42].

Where the target population and/or the clinical indication is not clearly specified, it is difficult for payers to control the diffusion of the intervention from populations where they have been proven to be beneficial into untested populations, a phenomenon known as indication creep. This was confirmed by the HTA experts whom we consulted, who see indication creep as a problem that may be more difficult to control for high-risk medical devices compared with medicinal products.

While QoL data is of key importance for patients, it is often not included as an outcome in trials. When QoL data are collected, they are often inadequate. Ideally, QoL should be measured using both a disease-specific instrument and a generic instrument (e.g. EQ-5D-5 L), at several time points over a sufficiently long period of time, enabling the calculation of quality-adjusted life years (QALYs) over a longer period. Inexplicably, results for QoL are sometimes considered confidential by the company that sponsored the trial [43]. Therefore, it is also necessary that the regulators at all levels and the payers/HTA agencies coordinate their recommendations on measuring and reporting QoL data.

Studies that do not meet the ethical requirements in the Declaration of Helsinki regarding the choice of comparator should be refused by ethics committees. Nevertheless, the MDR is not explicit regarding the comparator in clinical studies. From an HTA perspective, an RCT with a control group consisting of a cost-effective ‘standard care’ treatment, investigating patient-relevant outcomes is necessary to assess the added therapeutic benefit, if any, of the new device and to inform the calculation of its incremental cost-effectiveness ratio (ICER). The ICER is used for determining its comparative value and deciding whether it should be reimbursed or not. This applies to both medicinal products and medical devices. For high-risk medical devices, the lack of sound comparative clinical data hinders national reimbursement procedures [14, 40, 44]. Indeed, the lack of direct comparison with standard care in actual clinical practice and insufficient collection of long term patient-relevant outcomes, which payers need to make the reimbursement decision, have been reported to make such decisions difficult [42]. Comparative RCTs are also important for clinicians, so that they can make well-informed treatment choices and openly discuss them with the patient [45].

Ultrarare disorders may be an exception since the numbers are so small that it is unlikely that either RCTs or observational studies will be able to provide conclusive results. Nevertheless, randomization remains essential to balance any factors that influence outcomes besides the intervention (i.e. both the known and the unknown unknowns) across treatment groups, so as to minimize bias and justify any inferences being made. Observational study results that indicate that a device is efficacious may be contradicted when an RCT is performed, because well-designed RCTs minimize the influence of potential systematic biases and confounding factors [46]. Large observational data sets are, of course, useful for other purposes, such as the identification of long-term and rare adverse effects.

Representatives of the medical device industry have argued that conducting RCTs for medical devices is often not possible or ethical. However, healthcare payers need to assess new interventions to see if they have any added therapeutic benefit. RCTs with medical devices are feasible [47, 48]. HTA bodies have published study designs for medical devices that enable the generation of comparative evidence [49, 50]. Additionally, RCTs conducted in the context of pre-market approval for innovative high-risk devices in the US demonstrate that it is both ethical and possible to conduct RCTs on medical devices [47]. Also, in Europe, the device trials approved by a large ethics committee in Berlin show that a growing proportion of the device trials are randomized [10]. Furthermore, Neugebauer et al. (2017) also discuss the difficulties of conducting RCTs for medical devices and propose a number of solutions to support the generation of evidence using RCTs [48]. The proposed solutions include the use of core outcome sets, the blinding of assessors where needed, and the use of study designs that take learning curves into account.

Other frameworks for generating comparative evidence have been proposed for implantable medical devices, with attention to device-specific critical elements in trial design and conduct [5], or that also consider the effect that CE marking has on diffusion and uptake of new devices, as proposed by Neugebauer et al. (2010) [51].

### Transparency

According to Wild et al. (2014) there is a lack of transparency regarding how devices are approved and, particularly, the information assessed by Notified Bodies [27]. Sauerland et al. (2014) explains “When trying to find data on a single device or a group of medical devices, surgeons will often have difficulties in finding information, because preclinical and clinical data on medical devices may exist but are kept on file by the manufacturer.” [18].

There have been numerous calls and suggestions to rectify this. Fraser et al. (2018) [45] clearly demanded: “The evidence submitted by manufacturers when seeking approval of their high-risk devices must be publicly available, including technical performance and premarket clinical studies. Giving physicians access to this information supplements the peer-reviewed scientific literature and might be essential for comparing alternative devices within any class. Interested patients should be encouraged to review the evidence for any device that has been recommended for them.” Heneghan et al. (2017) suggest that one way to do this is to create and maintain a publicly accessible registry of all invasive medical devices, recording the necessary clinical evidence and details of marketing status when being placed on the market [37].

### Potential solutions

#### A stepwise introduction

Several groups, including KCE, have recommended a stepwise approach to introducing innovative medical devices based on evidence generated [18, 40, 41, 52]. The IDEAL (Idea, Development, Exploration, Assessment, Long-term study) framework, which also takes a stepwise approach, was developed by a group of surgical experts (the Balliol Colloquium) to improve surgical research and reporting of results [3, 4, 53–55]. Sauerland et al. (2014) warn that *all* the steps in the IDEAL framework need to be taken [18], including conducting RCTs to determine efficacy: “The pitfall to be avoided is acceptance of preliminary non-randomized data as proof of the new devices or procedure’s superiority.” This stepwise approach to introducing high-risk devices whilst gathering additional robust RCT data on their effectiveness and longer-term safety profile is another potential way of preventing the widespread adoption of unproven treatments. However, this requires the support of the regulatory system [29] to limit the risk of harm [56, 57]. Additional legislation may be required to allow only limited market access until this data is available and to require special reporting of all clinical outcomes post-CE marking. Currently, obtaining CE marking enables unrestricted access to the European market, whereas a graduated approach is needed to allow stepwise introduction based on the evidence generated.

#### More efficiency in clinical evidence generation

Comparative evidence during the pre-market clinical development can be generated more efficiently, reducing the cost and complexity of RCTs [58]. Pragmatic trial designs and factorial designs should be considered. One highly efficient study design providing possibilities for both pre- and post-market data collection concerns registry-based trials. Patient disease registries, particularly for rare diseases but also in other domains, could provide the infrastructure for highly efficient adaptive platform randomized trials, where standard care is compared with new interventions (devices or drugs) as they become available [59]. Governments should facilitate these efficiency gains by supporting clinical coding standardisation and providing an appropriate international legal framework and the associated information technology infrastructure. Furthermore, some authors have argued that an independent third party should run the comparative trials instead of the company that has developed or produced the device [60].

#### Focus on pre-market evidence generation or post-market use only in research

Depending on the device under consideration, a more controlled post-market ‘use only in research’ phase might be considered for reimbursement by payers. An important question is who should pay for new treatments whilst additional information is being collected as part of any post-market obligations? Publicly-funded trials [61] can also generate comparative data, but may, as with all post-market RCTs, face slow recruitment as patients and clinicians may have a strong preference for the newly marketed innovative device when being compared with usual care [3]. Even when demanded by regulators or by payers, the delivery of hard evidence for medical devices in the post-market period has been shown to be highly uncertain. As reported by Olberg et al. studies of high quality are seldom performed after the device is placed on the market [15]. This may lead to delays, often of many years, before clinicians and patients can make an informed treatment choice. The harms associated with not knowing what device is best for use in a specific patient are more difficult to estimate but are likely to be real and significant. Therefore, robust evidence generation should start in the pre-market phase.

#### Reinforcing post-market device safety surveillance

A remarkable number of CE marked high-risk devices have either been recalled or failed to obtain FDA approval [27, 35]. Given the reliance on post-market data collection for device safety, it is important to note that physicians who implant cardiovascular or orthopaedic devices may consider the reporting of adverse events with medical devices to be unnecessary, impossible, or pointless for various reasons [62, 68]. Thus, educational efforts emphasizing the importance of reporting may be needed for physicians.

#### Stimulate an early dialogue with device manufacturers

Multi-stakeholder initiatives, such as the EU-funded Coordinating Research and Evidence for Medical Devices project (CORE-MD), could help with starting a multi-stakeholder dialogue [63]. In addition, there is a need for early dialogues with the medical device companies, in order to improve the design of clinical investigations intended to support reimbursement decisions and manufacturers’ claims of added therapeutic benefit (or at a minimum, claims of clinical efficacy) [64–66]. Under the EUnetHTA 21 project, methods and processes are being developed and are being piloted for the JSC and JCA, set out in the HTAR, with the European HTA bodies [7]. These initiatives could result in better comparative evidence and greater transparency of the evidence for medical devices entering the EU market.

#### Common specifications on pre-market study design

It remains to be seen how the MDR will help companies and Notified Bodies in the generation of comparative evidence of the new device versus standard care treatment. Recent data from the German HTA body, IQWIG, show that for the first nine implants (May 2021 – March 2023) with an opinion provided under the CECP [19] only one manufacturer’s clinical evaluation contained an RCT, and it enrolled only a small sample of 43 patients [67], while 4 out of 9 were cardiovascular implants. More impact can be expected from the development of common specifications and enacting delegated acts that require the use of appropriate pre-market study designs to evaluate specific categories of medical devices.

#### Avoiding unwanted side-effects of the MDR

It is important to be aware of the unintended adverse consequences to some patients of implementing the MDR, as manufacturers may withdraw relatively cheap, and therefore, low profit, devices from the market rather than paying the additional costs that meeting the requirements of the MDR would incur. A limited number of these devices may be essential for a group of patients, thus as Melvin et al. (2022) point out [68], specific measures may need to be taken by the authorities to ensure their continued availability. In fact, partly due to this, the transition period has recently been extended, with full implementation of the MDR now pushed out until December 31, 2028.

### Key recommendations

Governments should invest in infrastructure to reduce the cost and improve the quality of medical device clinical trials, including the development of comprehensive patient registries. This will facilitate efficient designs that generate comparative evidence.

Common specifications should be developed regarding the most appropriate pre-market study designs for evaluating specific categories of medical devices, and their use required by legislation.

Studies that do not meet the ethical requirements in the Declaration of Helsinki regarding the choice of comparator should be refused by ethics committees.

The EU should explicitly state that clinical data underpinning the CE marking cannot be protected by confidentiality requirements and must be made publicly available.

HTA bodies and payers should not accept evidence that is too weak to come to meaningful conclusions on added therapeutic benefit.

Healthcare systems should institute pro-active post-market surveillance systems.

Clinicians and the public need to be educated about the lack of evidence for medical devices and the need for RCTs to demonstrate the effects of a treatment.

A more complete set of policy recommendations can be found in the KCE report [2].

## Conclusions

There is a huge lack of transparency regarding the clinical evidence underpinning the legal placement of medical devices on the European market. The little data that is available suggests that studies are of low quality and there is a heavy reliance on evidence from other devices. Sometimes this evidence is from devices that have been withdrawn or known to be unsafe or ineffective. This means that devices are entering the market without reliable evidence to determine if they are safe and effective, putting patients at serious risk of harm.

HTA bodies need RCTs to assess comparative effectiveness and added therapeutic value, without this, reimbursement decisions prove difficult and reimbursement may be delayed. The MDR does not sufficiently stimulate the generation of robust comparative evidence. The CE marking system for high-risk medical devices relies heavily on post-market data collection, yet this may take years and be too late to inform clinical decision-making for thousands of patients.

Therefore, the transparency of the clinical evidence provided to obtain CE marking must be improved. RCTs need to be conducted at the earliest stage possible, preferably in the pre-market stage, and the quality of these trials also needs to be improved. Pro-active and multi-faceted post-market surveillance activities also need to be developed and introduced to detect poorly performing devices, and patient registries could be used for conducting more efficient trials to assess comparative effectiveness.

## Data Availability

Not applicable, the material summarized in this paper is based on a published KCE report.
